# An overview on multimodal imaging for the diagnostic workup of pleural mesothelioma

**DOI:** 10.1007/s11604-023-01480-5

**Published:** 2023-09-07

**Authors:** Michela Gabelloni, Lorenzo Faggioni, Maria Chiara Brunese, Carmine Picone, Roberta Fusco, Giovanni Donato Aquaro, Dania Cioni, Emanuele Neri, Nicoletta Gandolfo, Andrea Giovagnoni, Vincenza Granata

**Affiliations:** 1 Nuclear Medicine Unit, Department of Translational Research, University of Pisa, Via Roma 67, 56126 Pisa, Italy; 2 Academic Radiology, Department of Translational Research, University of Pisa, 56126 Pisa, Italy; 3 Diagnostic Imaging Section, Department of Medical and Surgical Sciences and Neurosciences, University of Molise, 86100 Campobasso, Italy; 4 Division of Radiology, Istituto Nazionale Tumori IRCCS Fondazione Pascale-IRCCS di Napoli, 80131 Naples, Italy; 5 Medical Oncology Division, Igea SpA, 80013 Naples, Italy; 6 Diagnostic Imaging Department, Villa Scassi Hospital-ASL 3, 16149 Genoa, Italy; 7 Department of Radiology, University Hospital “Azienda Ospedaliera Universitaria Delle Marche”, 60126 Ancona, Italy; 8 Department of Clinical, Special and Dental Sciences, Università Politecnica Delle Marche, 60126 Ancona, Italy

**Keywords:** Pleural mesothelioma, Multimodal imaging, Staging, Radiomics

## Abstract

Pleural mesothelioma (PM) is an aggressive disease that has a strong causal relationship with asbestos exposure and represents a major challenge from both a diagnostic and therapeutic viewpoint. Despite recent improvements in patient care, PM typically carries a poor outcome, especially in advanced stages. Therefore, a timely and effective diagnosis taking advantage of currently available imaging techniques is essential to perform an accurate staging and dictate the most appropriate treatment strategy. Our aim is to provide a brief, but exhaustive and up-to-date overview of the role of multimodal medical imaging in the management of PM.

## Introduction

Pleural mesothelioma (PM) is a relatively rare, but deadly primary neoplasm of the mesothelial cells lining the pleura and, in a minority of cases, the peritoneum, pericardium or tunica vaginalis testis. Its characteristically aggressive biological behavior, associated with a tendency to spread along nearby tissues and to grow as multiple tumor deposits rather than as a single mass, makes it hard to treat despite the current availability of multimodal treatment approaches (including surgery with or without hyperthermic chemotherapy, radiation therapy, chemo- and immunotherapy).

Asbestos is a major causal factor of the disease, and long-lasting bans on its use in most industrialized countries have contributed to a gradual decline in PM prevalence compared to decades ago. Nevertheless, PM is still a relevant cause of cancer-related morbidity and mortality, due to the very long delay between asbestos exposure and disease onset, and to a lesser extent, to asbestos-unrelated factors [1].

Multimodal imaging plays a pivotal role in the diagnostic management of PM, enabling accurate disease detection and staging and guiding physicians to the choice of the best therapeutic strategy. In the workup of PM, improving diagnostic accuracy to detect even small neoplasms at earlier stages is paramount to maximize treatment effectiveness and attain oncologic radicality. Another goal is to refine the prediction of response to more specific, personalized therapies through the extraction of quantitative imaging biomarkers that correlate with tumor biology, clinical parameters, and treatment-related variables, optimizing patient progression-free survival (PFS) and overall survival (OS) and quality of life [2–4].

The purpose of this narrative review is to summarize the evidence collected so far on the role of modern imaging modalities in the diagnostic management of PM.

## Risk factors, pathogenesis and classification

According to the Global Cancer Observatory, mesothelioma ranks 32nd in terms of incidence among all cancers, with 30870 new cases being diagnosed worldwide in the year 2020 (for comparison, breast cancer ranked first with an incidence of 2261419 new cases). Of them, 13648 (44.2%) occurred in Europe, with a higher age-standardized incidence rate in males than in females (2.5 vs 0.46) [5]. The pleura is the commonest location of mesothelioma (84.9%), followed by the peritoneum (7.2%) and other locations, including the pericardium (0.1%) and the tunica vaginalis testis (0.2%) [6].

The great majority of PMs (reflecting the predominant pleural location of the disease) are related to asbestos exposure. Asbestos refers to a group of natural mineral fibers composed of silicates, which can be classified as amphiboles (crocidolite, amosite, tremolite, actinolite and anthophyllite) and serpentines (chrysotile) based on their chemical composition and fiber morphology. Chrysotile (also known as white asbestos) makes up 90–95% of all asbestos fibers used worldwide. Owing to its insulating properties and low cost-effectiveness, asbestos used to be incorporated into a wide variety of materials and products, such as cement, tar, and brake pads.

Long-term exposure to asbestos fibers is a known causal factor for PM, with asbestos workers being at highest risk for developing the disease. In the United States, asbestos bans between 1973 and 1989 have paved the way to a gradual decrease in PM incidence and mortality rates two decades later. In the European Union, the use of asbestos has come to a complete stop in 2005, with national bans having been enforced in several member states well before [7, 8].

The hazards related to asbestos exposure can extend outside the workplace, such as for workers operating close to asbestos-contaminated areas and exposed to airborne fibers (e.g., in the shipbuilding industry). Living near a facility that uses asbestos, such as mines or manufacturing plants, is associated with a higher risk of developing PM [7]. Family members of asbestos workers can also be at risk by exposure to fibers brought home on the worker’s clothing. While asbestos may not be actively used and marketed, built-in asbestos inside commonly used objects (including pipes, insulation, stoves, heating devices, asbestos sheeting and roofing) may pose a hazard to people in contact with or living near them [8].

Inhalation is the predominant exposure route to asbestos fibers. Fibers narrower than 3 µm and shorter than 5 µm may be inhaled and end up in the pleura, where they trigger a chronic inflammatory response that ultimately leads to a loss of tumor-suppressive mechanisms. There is increasing evidence of a genetic basis underlying a higher susceptibility to asbestos carcinogenesis. Carriers of *BAP1* mutations have a higher incidence of malignancies related to a cancer syndrome including PM, in contrast to unaffected family members [9, 10].

The latency time between asbestos exposure and PM onset can be as long as approximately 40 years on average, although it may extend to 60–70 years. On this basis, it may be hypothesized that although the earliest asbestos bans date back to approximately 50 years ago, we might be just now beginning to see the beneficial effects of such bans on public health [8, 11]. A recent article investigating global trends in PM diagnosis from 1990 to 2017 showed that PM incident cases and age-standardized incidence rates have begun to decrease after 20 years of complete asbestos ban, resulting in a lower PM incidence in patients younger than 50 years, paralleled by a higher incidence in patients older than 70 years and in countries with a low socio-demographic index [12].

Several molecular mechanisms are involved in the development of asbestos-related PM. Following asbestos exposure, macrophages fail to phagocytize asbestos fibers and release reactive oxygen and nitrogen species (ROS/RNS) that recruit other inflammatory cells and promote genotoxic damage. Repeat DNA damage to mesothelial cells may induce multiple oncogenic mutations of genes involved in DNA repair (*BAP1/TP53*), the Hippo pathway (*NF2* and *LATS1/2*), the mTOR pathway (*TSC1/2* and *PI3K*), DNA methylation (*SETD2*), and cell cycle control (*CDKN2A/B*) [10, 13]. Inflammatory mediators promote cell survival and mesothelioma growth. Uncontrolled regulation of epithelial to mesenchymal transition also plays a key role in the pathogenesis, tumor progression, and immune system evasion of PM, forming the basis for several types of target therapies.

Based on the 2021 WHO Classification of Tumors of the Pleura and Pericardium, mesotheliomas can be classified as localized or diffuse and by definition are all malignant, since the very rare condition formerly known as well-differentiated papillary mesothelioma (WDPM, consisting of papillary formations covered by a single layer of bland mesothelial cells without stromal invasion) has been renamed as WDPM tumor (WDPMT) owing to its relatively indolent behavior, to differentiate it from diffuse PM [14]. Another update from the 2015 WHO classification is represented by the recognition of mesothelioma in situ (MIS) as a form of preinvasive mesothelial tumor, which is characterized by a single layer of relatively bland mesothelial cells growing along the pleural surface with specific genetic alterations (loss of *BAP1* or *MTAP*, homozygous deletion of *CDKN2A*) and can be associated with recurrent unilateral pleural effusions of unknown origin, without tumor evidence at thoracoscopy or imaging. Among invasive PMs, localized PM typically presents as a solitary localized mass at imaging, surgery and histopathology (without invasion beyond its circumscribed tumor borders) and carries a more favorable prognosis than diffuse PM when completely resected. Conversely, diffuse PM is characterized by diffuse pleural involvement and invasion of adjacent structures (i.e., fat, skeletal muscle and/or lung parenchyma), and from a histopathological viewpoint, it can be classified as epithelioid (round epithelioid cells, accounting for about 60% of cases), sarcomatoid (spindle cells, making up for approximately 20% of cases), and biphasic (or mixed, including epithelioid and sarcomatoid components) [14, 15]. Common immunohistochemical markers for PM diagnosis include calretinin, CK5/6, WT1, and D2-40. Sarcomatoid PMs may contain osteoid or chondroid elements, which can be detected as intratumoral calcifications at diagnostic imaging [15].

## Imaging diagnosis: which modality?

### Chest radiography

**Fig. 1:**
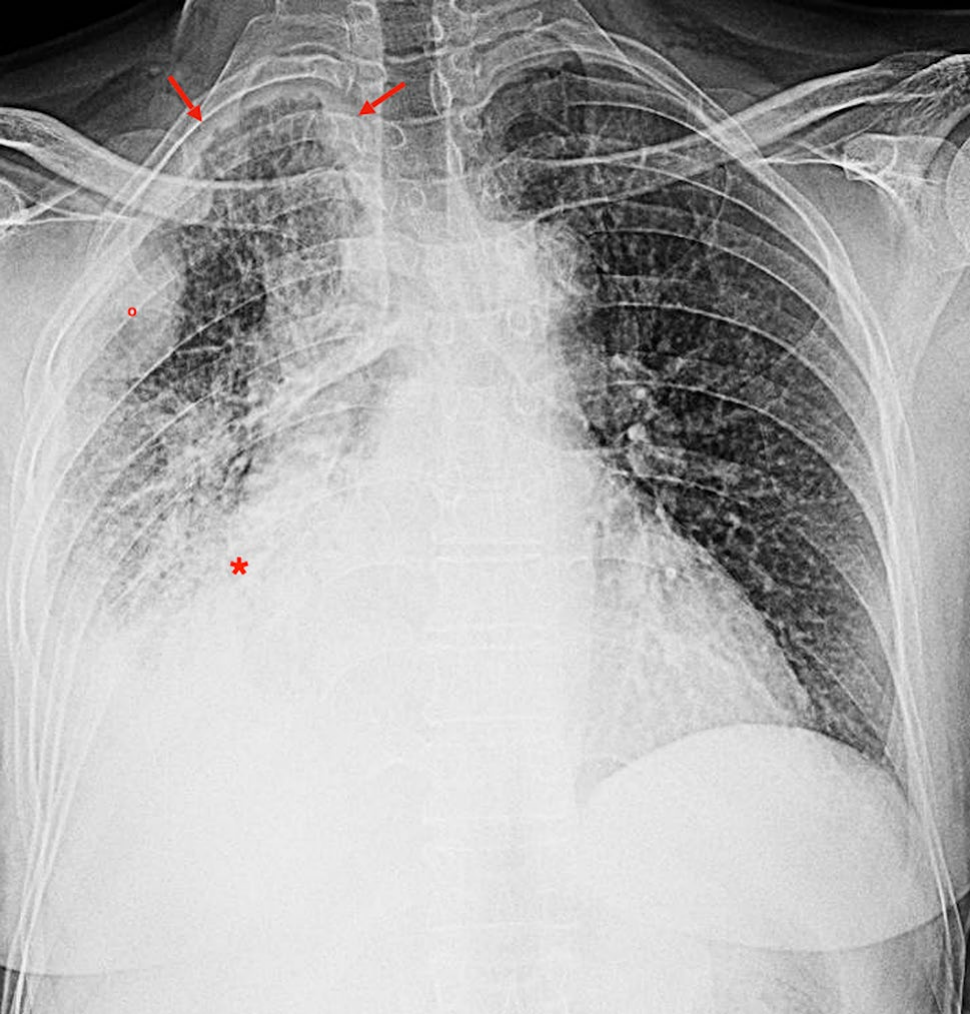
74-year-old man with history of occupational asbestos exposure and shortness of breath. Chest radiograph (frontal view) shows right-sided pleural effusion with consolidation of adjacent lung parenchyma (asterisk), pleural opacities (circle) and pleural thickening (arrows). Right lung volume loss and ipsilateral mediastinal enlargement can also be seen

Chest radiography represents the first-level imaging modality for the detection of PM. According to the International Mesothelioma Interest Group (iMIG) Pleural Imaging Expert Panel, both frontal and lateral chest radiographs should be obtained taking care to include anterior and posterior costophrenic angles [16]. A unilateral pleural effusion is the most typical finding at presentation, occurring in a variable proportion of patients (ranging from 30 to 80%). A pleural-based mass without pleural effusion can be seen in less than 25% of patients, whereas diffuse pleural thickening or extensive lobular pleural-based masses are present in about half of cases (Fig. 1). Pleural plaques are thickened areas of parietal pleura made up of connective tissue that can become calcified, and represent the commonest radiographic manifestation of long-standing asbestos exposure, occurring in about 20% of cases [2]. However, radiographic findings are often nonspecific and vary depending on disease stage at diagnosis, prompting the use of second-level imaging tests to accurately assess disease burden and differentiate PM from nearby anatomic structures and other conditions [16].

### Computed tomography

Multidetector computed tomography (CT) is the mainstay for the morphologic assessment of PM and is paramount for primary staging and treatment planning. The ability of modern CT scanners to acquire images with submillimeter spatial resolution and voxel isotropy in a few seconds allows to reliably depict the thoracic and abdominal anatomy of interest in any imaging plane, improving the assessment of subtle findings and complex anatomies (such as the diaphragm, pericardium or peritoneal surfaces) [17–20]. Since PM often has a multiplanar growth pattern with discontinuous lesions of non-spherical shape across multiple CT sections, the iMIG Pleural Imaging Expert Panel recommends that multidetector CT images be interpreted in three imaging planes with a high spatial resolution (typically 1–2 mm slice thickness for axial images and 2–3 mm for sagittal and coronal views). while multidetector CT lends itself to quantitative measurements of disease burden over large body areas in a repeatable and relatively operator-independent manner, the peculiar growth pattern of PM can complicate the use of morphological response evaluation criteria in solid tumors (RECIST) at baseline and follow-up imaging. This warrants the adoption of modified response criteria (mRECIST 1.1) for the assessment of PM treatment response, implying that pleural measurements be made at consistent sites with clinical relevance (up to six across no more than three sections at least 1 cm apart), preferably above the left atrium and below the aortic arch to maximize reproducibility [21].

**Fig. 2:**
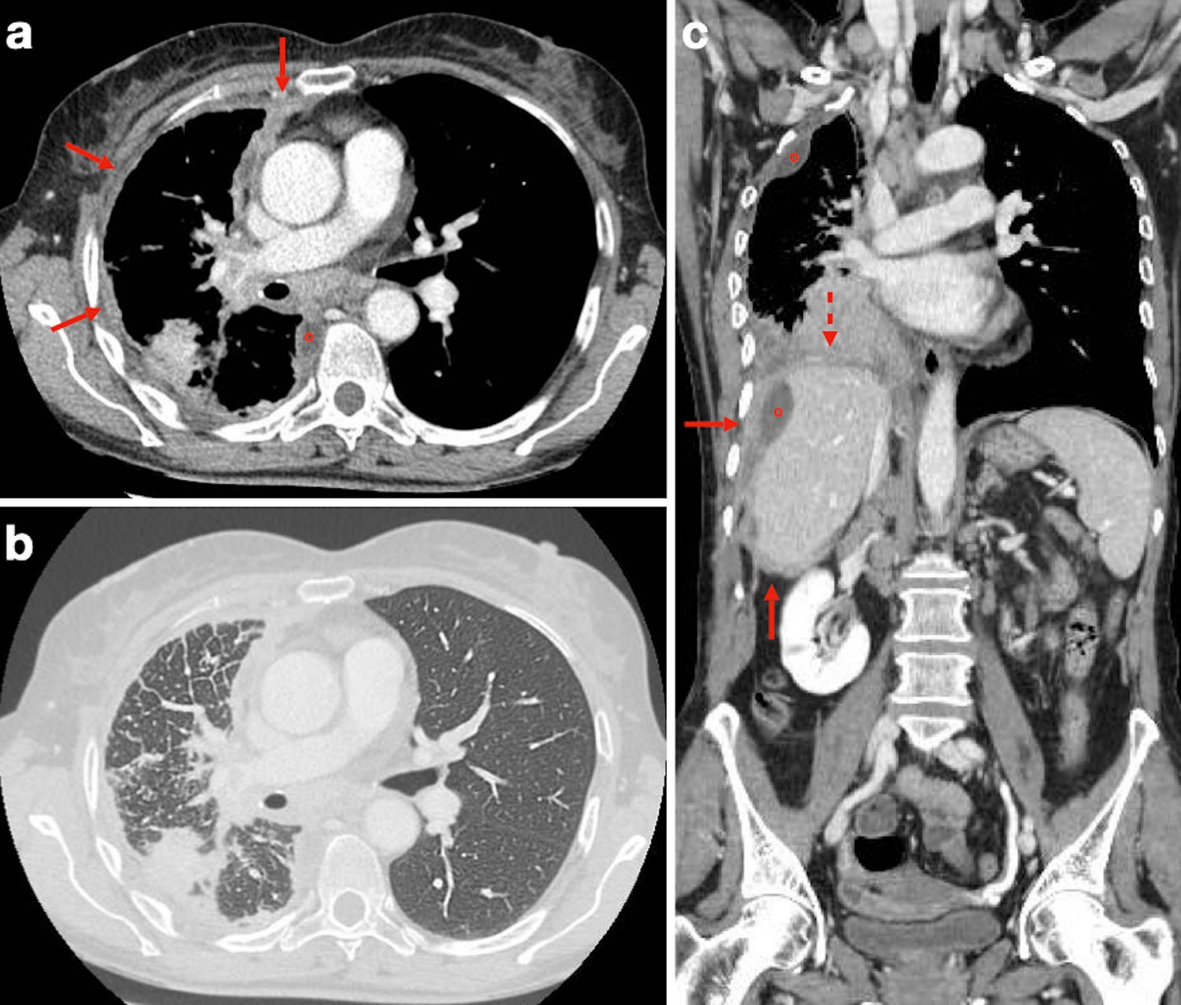
Same case of Fig. 1. **a** Multidetector CT examination shows diffuse, irregular pleural thickening with contrast enhancement and infiltration of the anterior mediastinal fat (arrows), loculated pleural effusion (circle) and volume loss of the right lung. **b** Image reconstructed with lung window settings and kernel shows diffuse thickening of the interlobular septa of the right lung with some micronodular aspects, consistent with lymphangitic carcinomatosis. **c** Coronal view reveals large mass at the right lung base with infiltration of the ipsilateral hemidiaphragm (dashed arrow). Tumor tissue extends along the Glissonian capsule of the liver (arrows). Loculated pleural and perihepatic effusions (circles) can also be seen, as well as mediastinal and supraclavicular lymphadenopathies

The scan volume should encompass the entire chest with a caudal extension to the level of L3 to ensure coverage of the entire posterior costophrenic angles, and intravenous administration of iodinated contrast material can greatly improve CT detection and quantification of PM deposits and should always be performed (unless clinically contraindicated) with a typically recommended scan delay of 50–60 s after the beginning of contrast medium injection. An additional CT series with a sharp reconstruction kernel should also be generated for the evaluation of the lung parenchyma and bones [16] (Fig. 2).

Pleural thickening can manifest in up to 92% of patients and is highly variable in extent, thickness, and degree of nodularity. The CT finding of contrast-enhancing pleural thickening larger than 1 cm, involving the mediastinal, parietal and/or fissural pleura with a nodular, lobular or circumferential pattern, is highly suggestive of PM. Pleural effusions and plaques can be encountered in approximately 75–20% of cases, respectively. Circumferential pleural thickening associated with rind-like encasement of the lung parenchyma and ipsilateral pulmonary volume loss can be seen in late-stage disease. Calcified pleural plaques can be found in approximately 20% of PM patients and may become engulfed by the primary tumor. The affected hemithorax is often contracted with ipsilateral mediastinal shift, narrowed intercostal spaces, and elevation of the hemidiaphragm.

**Fig. 3:**
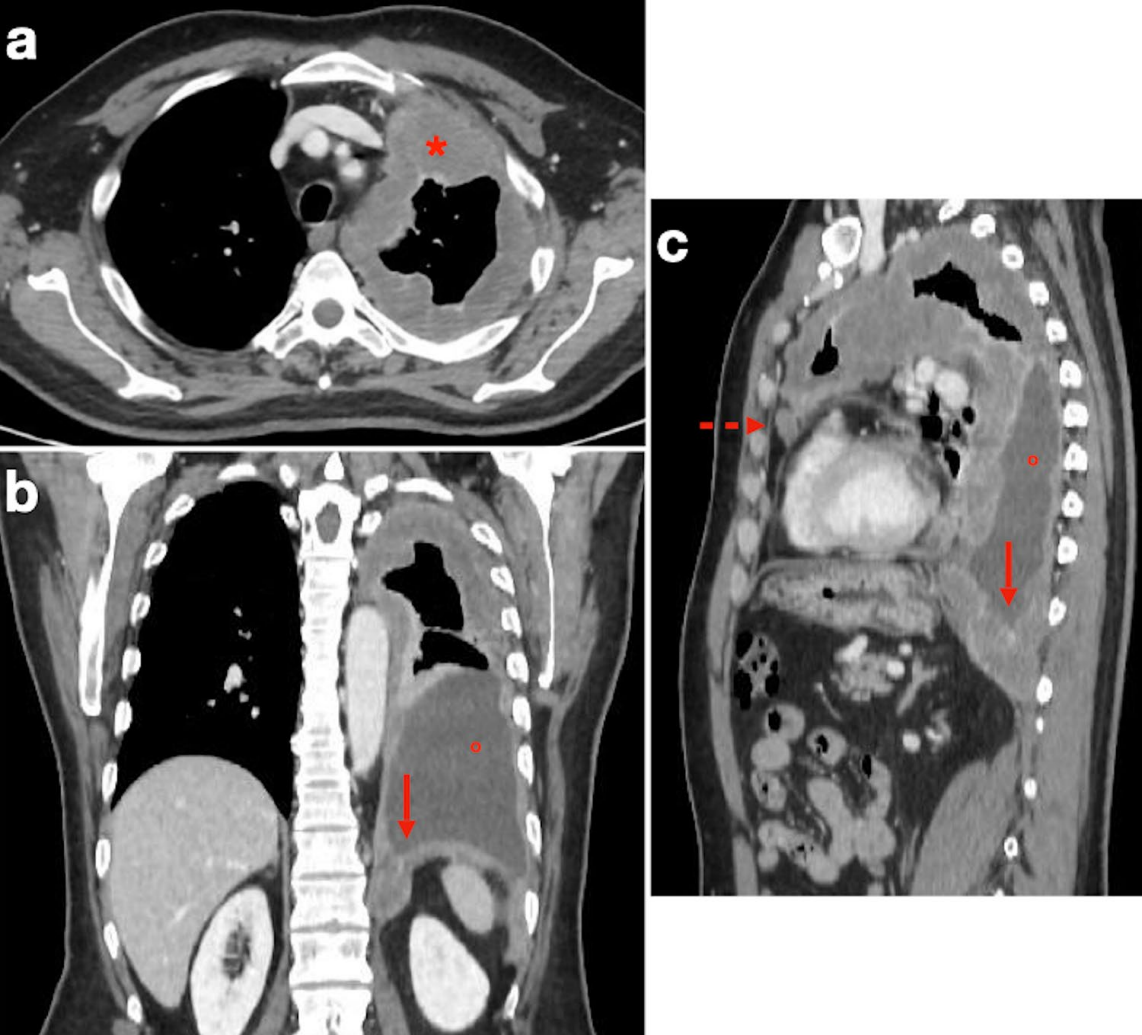
72-year-old man with history of asbestos exposure undergoing multidetector CT examination for PM staging. **a** Axial image shows diffuse, rind-like soft tissue with contrast enhancement that determines marked pleural thickening and left lung encasement (asterisk). **b** Loculated pleural effusion (circle) is present, along with infiltration of the ipsilateral hemidiaphragm (arrow). **c** Infiltration of the epicardial fat (dashed arrow) with no clear demarcation from the pericardial layer can also be seen

Tumors with multifocal or diffuse invasion of the chest wall, mediastinal structures, spine or pericardium, and/or with involvement of the contralateral pleura, transdiaphragmatic extension or metastatic disease are considered unresectable. Contrast-enhanced CT can help assess locoregional PM involvement by revealing pericardial, mediastinal, tracheal, esophageal and/or chest wall infiltration, as well as transdiaphragmatic invasion of abdominal organs (Fig. 3). Pericardial involvement can manifest as pericardial thickening and/or effusion. Loss of fat planes between mediastinal structures is a sign of mediastinal invasion, and encasement of more than 50% of the circumference of the esophagus or trachea suggests infiltration of these organs. Likewise, cancellation of normal extrapleural fat planes, infiltration of intercostal muscles, rib displacement or bone destruction are suggestive of chest wall invasion. Occasionally, seed implantation of PM to the chest wall can occur via needle biopsy tracks, surgical scars, and chest tube tracts.

**Fig. 4:**
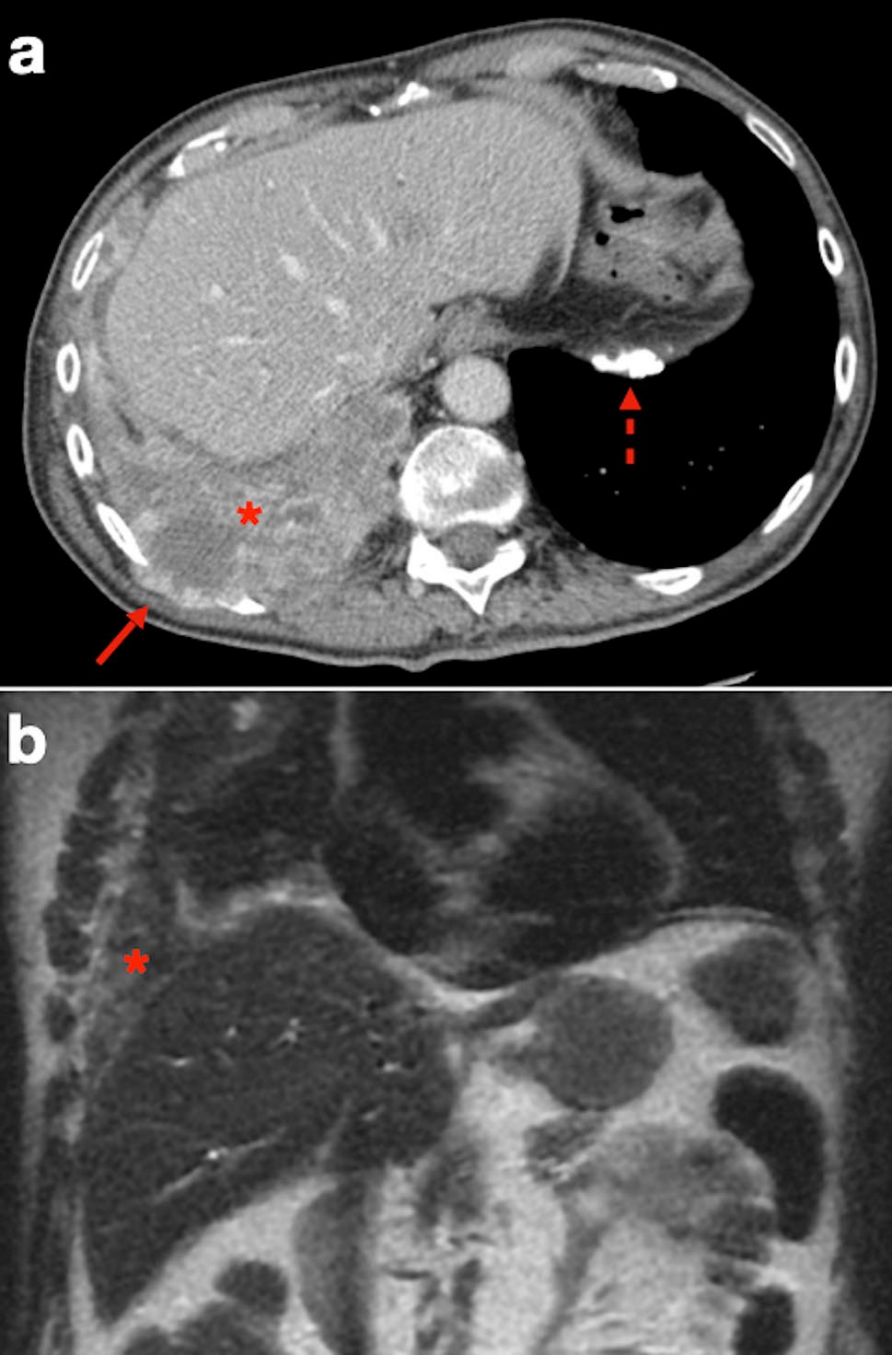
69-year-old man with history of asbestos exposure and locally advanced PM. **a** CT image shows large soft tissue mass (asterisk) that cannot be separated from the right hemidiaphragm. Infiltration of the posterior chest wall is also present (arrow). Note left-sided calcified diaphragmatic pleural plaque (dashed arrow) related to long-standing asbestos exposure. **b** Coronal T2-weighted MR image shows inhomogeneously hyperintense tissue based on the right diaphragmatic pleura with cancellation of the nearby subdiaphragmatic fat plane (asterisk)

While thickening of a hemidiaphragm is a common finding, CT has limited accuracy in evaluating transdiaphragmatic PM involvement, especially in more subtle cases. A hallmark of transdiaphragmatic spread is a soft tissue mass with contrast enhancement encasing the hemidiaphragm (Fig. 4). In contrast, a clear fat plane between the diaphragm and adjacent abdominal organs, together with a smooth diaphragmatic contour, suggests that the tumor is confined to the chest.

Lung metastases from PM can be detected on CT as parenchymal nodules and masses and, rarely, as diffuse miliary nodules. Albeit seldom, CT may also demonstrate extrathoracic spread of PM as direct liver invasion, retroperitoneal extension or retrocrural adenopathy.

Multidetector CT is the commonest modality for the evaluation of lymph node involvement. However, its accuracy remains suboptimal because lymph node enlargement alone does not prove metastatic nodal spread [2, 15]. While mediastinal lymph nodes with a short axis of 10 mm or more are considered abnormal based on CT morphological criteria, no specific size criteria have been established for other thoracic lymph nodes (such as internal mammary, retrocrural, and extrapleural ones), which should therefore be marked as abnormal if detected, regardless of their size [22].

**Fig. 5:**
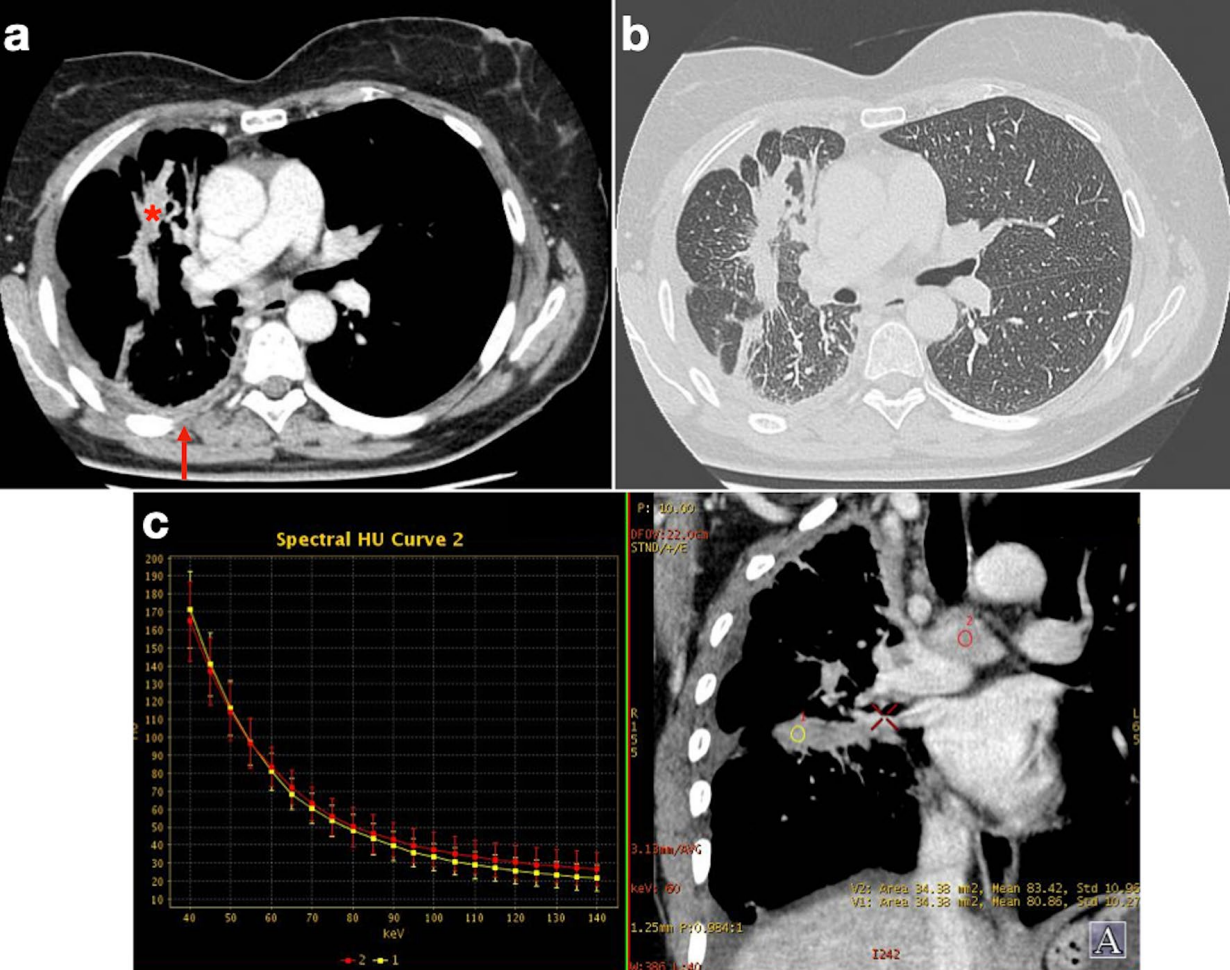
50-year-old woman with history of heavy smoking and no past asbestos exposure presenting with cough and right-sided chest pain. **a**, **b** Dual energy CT examination shows irregular pleural thickening with contrast enhancement (arrow) extending to the fissural pleura (asterisk), together with ipsilateral pulmonary volume loss. **c** Mediastinal lymphadenopathy is also present (red circle) with spectral curve comparable to fissural thickening (yellow circle). CT-guided biopsy revealed BRAF-mutated non-small cell lung carcinoma with predominant pleural involvement

CT can provide clues for differentiating malignant from benign pleural disease. The presence of pleural calcifications may suggest a benign process and may be a sign of asbestos exposure, but not a precursor of PM. Conversely, non-calcified PM can encase calcified plaques and mimic calcified tumors. Spectral CT can be helpful in directly measuring the iodine content of contrast-enhancing pleural masses, and Lennartz et al. showed that it can outperform conventional CT in discriminating pleural carcinomatosis from noncalcified benign pleural lesions with a sensitivity and specificity of 96% and 84% (vs 83% and 63%, respectively; *p* < 0.001) [23]. Diffuse pleural thickening and effusion can also result from conditions other than PM (e.g., asbestosis, infections, and other malignant pleural and extrapleural tumors) (Fig. 5), which can render CT-based diagnosis a challenging task [24].

### Magnetic resonance imaging

Thanks to its superior soft tissue resolution, chest MRI can be more sensitive than CT for evaluating the local invasion of the chest wall, diaphragm, and cardiovascular structures, and for differentiating malignant from benign pleural disease. Similar to multidetector CT, the iMIG Pleural Imaging Expert Panel recommends a thin-slice image acquisition protocol on three imaging planes using fat-suppressed pre- and post-contrast sequences (especially for the evaluation of trans-diaphragmatic invasion), coupled with axial T1-weighted images and axial and coronal T2-weighted images as recommended minimum image set [16].

**Fig. 6:**
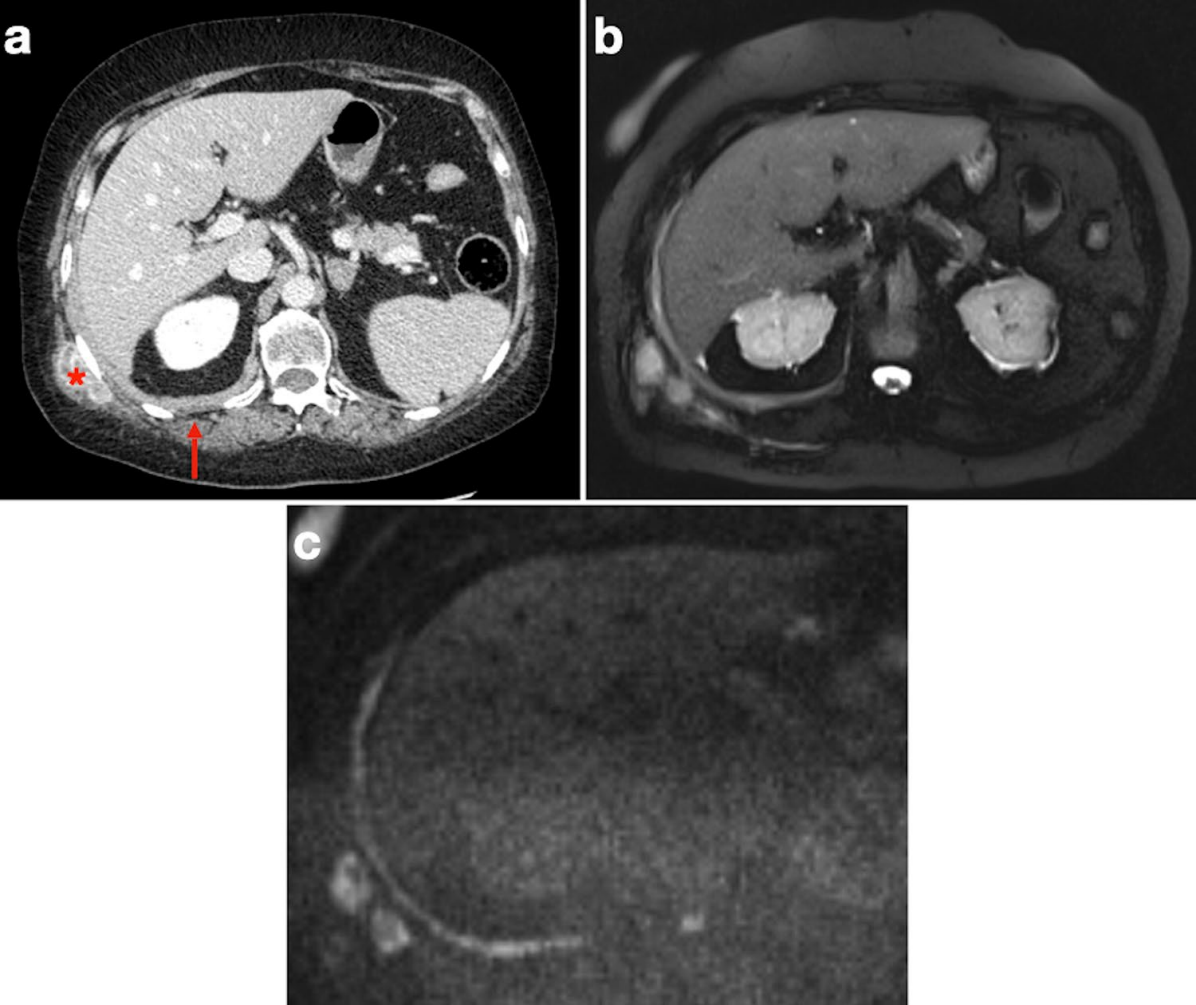
70-year-old man with history of asbestos exposure and known PM under chemotherapy. **a** Axial CT image shows irregular thickening with contrast enhancement of the posterior costophrenic sulcus (arrow) and nodular mass along the outer aspect of the right tenth rib, as a result of tumor seeding from previous percutaneous biopsy. **b** Axial fat-suppressed T2-weighted and **c** diffusion-weighted images clearly depict tumor deposits with sharp tumor-to-normal-tissue contrast

MRI features of diffuse PM include mildly T1-isointense or T1-hypointense, moderately T2-hyperintense, nodular pleural thickening or pleural masses, often associated with pleural effusion, which show contrast enhancement after intravenous administration of gadolinium-based contrast material. In patients with resectable disease, MRI can yield additional staging information, enabling a more accurate assessment of tumor extension and improved prediction of tumor resectability. Contrast enhanced T1-weighted fat suppressed images have been found to be the most reliable for detecting tumor spread into interlobar fissures and neighboring organs. While dynamic contrast-enhanced MRI (DCE-MRI) can allow the determination of perfusion parameters that reflect PM neoangiogenesis, diffusion-weighted imaging (DWI) may help distinguish between different histotypes and aid in post-treatment assessment (Fig. 6). No agreement has been found so far on the optimal apparent diffusion coefficient (ADC) cutoff for differentiating lesions, yet different strategies regarding the positioning of regions of interest (ROI) inside PM tissue have been explored in an attempt to minimize errors in ADC measurements [16, 25–27].

A major downside affecting the diagnostic quality of MR images is represented by artifacts, which can be of several kinds (e.g., susceptibility, aliasing, and motion artifacts). Artifacts related to chest motion can be minimized by means of electrocardiographic (ECG)-gating and respiratory compensation techniques. Especially the detection of subtle imaging findings on MRI is strongly dependent on a clear depiction of the pleura and nearby structures, which mandates optimal rejection of motion artifacts to ensure a reliable diagnosis.

### Positron emission tomography

**Fig. 7:**
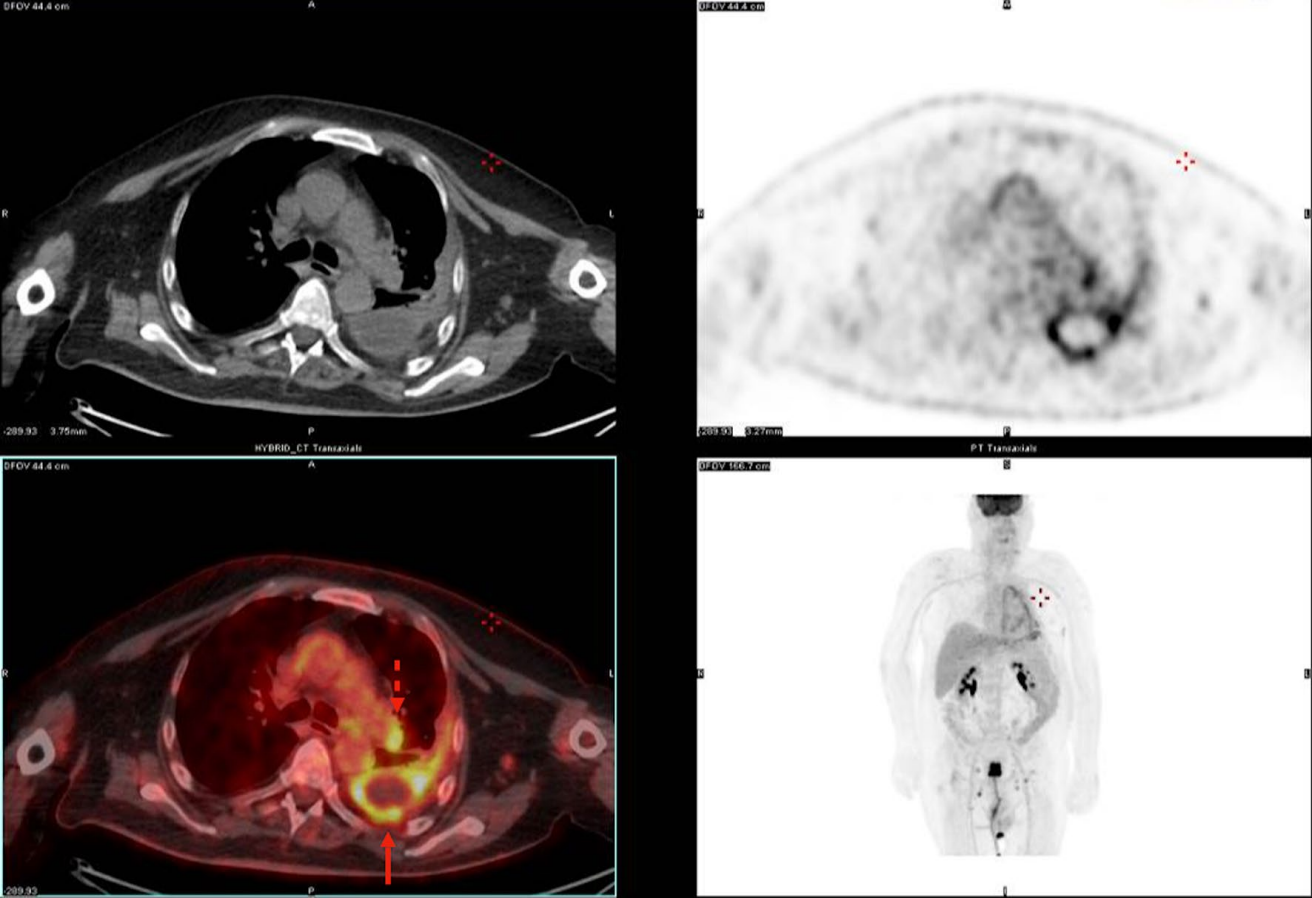
63-year-old man undergoing ^18^F-FDG PET/CT examination for PM staging. Pleural thickening (arrow) surrounding an area of loculated pleural effusion can be seen, along with ipsilateral hilar lymphadenopathy (dashed arrow)

^18^F-fluoro-deoxy-glucose (^18^F-FDG) PET is a functional imaging modality used for the evaluation of glucose metabolism in a broad spectrum of diseases. Like many types of cancers, PM cells exhibit a higher glucose consumption than healthy tissues due to increased glycolysis. Therefore, ^18^F-FDG PET typically shows avid glucose uptake (higher than in benign pleural diseases) in PM tissue, which can be helpful for the detection of occult metastases in lymph nodes and other sites (Fig. 7). Some authors have applied a SUV_max_ cut-off from 2.0 to 3.5 to differentiate PM from benign conditions, such as pleuritis of either tubercular or non-tubercular etiology [28]. However, pleural involvement could also occur in malignancies other than PM (such as thymomas, sarcomas and metastatic tumors of extrapleural origin), underscoring the need for pathological confirmation via video-assisted thoracoscopy (VATS) or CT-guided biopsy.

Patients with PM may have diffuse pleural thickening (due to extensive fibrosis as a result of long-term asbestos-related inflammation), but only focal areas of malignancy, for which ^18^F-FDG PET/CT can be decisive for disease detection and choosing the most appropriate biopsy site. Moreover, ^18^F-FDG PET/CT should be performed before any surgical intervention (such as VATS) implying talc pleurodesis, because the latter can cause a massive pleural ^18^F-FDG uptake as a result of an intense inflammatory reaction that may last for decades [28]. The iMIG Pleural Imaging Expert Panel recommends that in order to avoid false positive findings, ^18^F-FDG PET/CT for restaging should be deferred for a minimum of 6–8 weeks after surgery, with longer intervals of 8–12 weeks after thoracic radiation [16]. Overall, ^18^F-FDG PET/CT and ^18^F-FDG PET/MRI (as well as whole-body MRI) can yield a higher diagnostic accuracy for the TNM staging of PM than morphologic imaging based on CT and contrast-enhanced MRI [29]. Another study conducted on a small patient sample (*N* = 10) showed that ^18^F-FDG PET/MRI is feasible for clinical PM staging and can allow a more accurate loco-regional staging than PET/CT, especially T staging [30].

^18^F-FDG PET is a powerful diagnostic tool not only for the primary staging of PM, but also for the evaluation of treatment response and the detection of disease recurrence. Based on their morphology, four different patterns of PM relapse can be identified on ^18^F-FDG PET/CT: focal, linear, mixed (focal/linear), and encasing. The assessment of treatment response can be challenging, especially in case of limited signs of disease recurrence that may go undetected or misinterpreted on conventional morphologic imaging. The typical rind-like distribution pattern of PM has spurred the adoption of modified RECIST criteria instead of conventional, morphology-based treatment response criteria [28]. Owing to its ability to assess metabolic rather (and earlier) than morphologic changes after therapy, ^18^F-FDG PET/CT plays a pivotal role in evaluating response in PM patients undergoing systemic therapies, by means of metabolic response criteria such as EORTC (European Organization for Research and Treatment of Cancer) and PERCIST (PET Response Criteria in Solid Tumors) criteria, and/or semiquantitative and volumetric parameters, including metabolic tumor volume (MTV) and total lesion glycolysis (TLG). EORTC and PERCIST criteria have been found to be more accurate than mRECIST criteria for evaluating tumor response to chemotherapy and predicting prognosis in patients with unresectable PM [31], and ^18^F-FDG PET metabolic volume response can predict survival in PM patients receiving high-dose pembrolizumab [32].

As with talc pleurodesis, inflammatory reactions secondary to radiation therapy or immune-related adverse events during immunotherapy with immune checkpoint inhibitors can be misleading, demanding special care in the interpretation of ^18^F-FDG PET/CT findings in such conditions. Early-stage disease, epithelioid histology, and peritoneal involvement tend to exhibit lower ^18^F-FDG uptake, complicating discrimination between malignant and benign conditions.

To overcome the aforementioned limitations, other tracers than ^18^F-FDG have been investigated. ^11^C-methyl-methionine (MET), a radiolabeled amino acid, can be exploited due to the overexpression of L-Type amino acid transporter (LAT) in PM cells, reflecting increased tumor metabolism compared to other tissues. Some studies [33–35] have shown that LAT1 is overexpressed in approximately 50% of patients with PM, and have proposed ^11^C-MET PET/CT as an alternative imaging technique in PM patients with ambiguous findings on ^18^F-FDG PET/CT.

Another tracer with a potential role in the diagnostic workup of thoracic malignancies (including PM) is ^11^C-choline, a marker of phospholipid metabolism and cellular membrane turnover [36, 37]. Recently, the promising results from radiolabeled fibroblast activation protein-specific inhibitor (FAPI) compounds in various cancer types (particularly for histotypes with reduced glycolytic metabolism) have driven research to compare the diagnostic performance and theranostic potential of ^68^Ga-FAPI-04 versus ^18^F-FDG in patients with peritoneal mesothelioma. Due to the overall poor prognosis of mesothelioma (especially in advanced stage or recurrent disease), the demonstration of a clinical benefit for diagnostic and therapeutic FAPI agents could be a game changer for patient outcome [38].

## TNM staging

Because OS of PM patients primarily depends on the extent of the primary tumor (T status) and on lymph node involvement (N status), the iMIG has proposed the following tumor-node-metastasis (TNM) staging system for PM to guide treatment planning:

- Patients with early disease (T1), who may benefit from surgery with a curative intent.
- Patients who may benefit from surgery but may not necessarily be cured (T2 and T3).
- Patients for whom surgery may yield no benefit because of short OS and extensive local tumor spread (T4), and those with extensive regional node involvement (T4-N1, T4-N2) or distant metastases (M1), for whom surgery is not an option.

Distinguishing T4-stage disease (which defines locally advanced, technically unresectable tumor) from lower stages is mandatory to assess resectability. As mentioned above, invasion of mediastinal soft tissue masses without preservation of fat planes indicates unresectability. A soft tissue mass encasing the diaphragm is also considered unresectable [24]. According to AJCC prognostic groups and NCCN guidelines, surgical evaluation is indicated in patients with clinical stage I-IIIA (corresponding to TNM stages T1N0M0 through T3N1M0) and epithelioid histology following thoraco-abdominal staging by contrast-enhanced CT and pathological diagnosis. Such evaluation involves PET-CT (recommended before pleurodesis, and considering assessment by a multidisciplinary team with expertise in PM) and optional contrast-enhanced chest MRI for further analysis of possible chest, spinal, diaphragmatic, or vascular involvement based on CT imaging [39].

The main strengths of the various imaging modalities in the diagnostic work-up of patients with PM are summarized in Table 1.

**Table 1:** Main strengths of imaging modalities in the diagnostic management of PM

	Main strengths in diagnostic work-up of PM
Chest radiography	• Wide availability, low cost, very fast and simple image acquisition, low radiation exposure, easy repeatability • Often used for screening and/or as first imaging modality in patients at risk for PM (typically with pleural calcifications or known asbestos exposure), for follow-up under therapy (pleural effusion), detection of complications (e.g., pneumothorax, pneumonitis), and surveillance in case of suspected recurrence
CT	• Mainstay for morphological PM staging and follow-up • Excellent spatial resolution (allowing for multiplanar image assessment), good contrast resolution, fast and reproducible image acquisition, quantitative imaging • Good panoramicity with simultaneous assessment of pleura, soft tissues, lung parenchyma, vessels and bones • Can help differentiate PM (primary or recurrent) from mimickers (other tumors or non-neoplastic conditions) and detect post-therapy complications (e.g., post-surgery), dictating the subsequent management
MRI	• Good spatial resolution, excellent soft tissue contrast resolution, no radiation exposure, native multiplanarity • Can be useful in patients in whom administration of iodinated contrast material is contraindicated • Can assist surgical decision making and local staging in the presence of equivocal CT findings (e.g., for the detection of suspected chest wall invasion, mediastinal and diaphragmatic infiltration, as well as adrenal, neuroforaminal, epidural or brachial plexus involvement), and guide localized palliative radiation therapy • Advanced MR imaging techniques (DCE-MRI, DWI) can aid in the detection of disease recurrence and treatment planning
PET-CT	• Can be used to differentiate benign from malignant pleural disease (including post-treatment sequelae from recurrent PM), facilitate focused image-guided biopsy, and detect occult metastasis (especially extrathoracic and nodal disease spread) • Can provide an early assessment of therapy response, owing to its ability to detect metabolic rather than morphological changes • Allows semiquantitative and quantitative assessment of various pathways of disease metabolism [16]

## Radiomics in PM

Radiomics is an emerging methodology that holds promise in the advanced management of several disease conditions, with cancer being one of the areas where research is most active. Using artificial intelligence (AI)-based algorithms, images obtained with conventional protocols can be analyzed to extract a large amount of quantitative parameters based on mathematical models, reflecting characteristics of imaging data (so-called radiomics features) that go beyond visual image assessment and can be correlated with clinical, biological and other “omics” data. This approach can improve the diagnostic workup and prognostic assessment of cancer patients, allowing to predict early treatment response and avoid undue over- or undertreatment depending on the biological aggressiveness of each patient's specific disease, paving the way to a more individualized care [40–61]. Compared to conventional biopsy, radiomics has the advantage to capture the tumor tissue in its entirety instead of in limited areas of it, to guide biopsy towards specific tumor sites, and to be repeatable at follow-up imaging examinations [62].

To our knowledge, a few studies have investigated the role of radiomics in the management of PM patients so far. Pavic et al. [63] retrospectively evaluated the prognostic ability of 780 radiomics features extracted from staging ^18^F-FDG PET/CT examinations performed in 72 PM patients subsequently treated with curative surgery. PET-derived radiomic features showed a good predictive performance for PFS (mean concordance index of 0.67 in the training cohort and 0.66 in the internal validation cohort), and the PFS advantage with the PET radiomic model was associated with better OS in the low-risk group, whereas SUV_max_ and SUV_mean_ were not prognostic in terms of PFS and OS. Xie et al. [64] evaluated the predictive performance of radiomics to predict *BAP1* mutation status in PM patients using non-contrast CT images. The radiomics model based on three-dimensional ROI segmentation showed a good predictive performance for *BAP1* status in the training set (mean AUC 0.786, sensitivity 91.1%, specificity 61.7%, accuracy 74.3%) and test set (mean AUC 0.768, sensitivity 71.4%, specificity 75.0%, accuracy 73.9%), with a corrected AUC after 1000-times bootstrapping in the training set of 0.77 (95% confidence interval: 0.633–0.911).

## Conclusions

Despite bans on asbestos use, PM is likely to remain a relevant healthcare issue for years to come due to its very slow onset and difficult treatment. A multimodal diagnostic workup based on an appropriate usage of the various morphologic and functional imaging techniques is essential to ensure an accurate and timely PM staging and detection of disease recurrence.
